# Microcrystalline Cellulose-Blended Polyethersulfone Membranes for Enhanced Water Permeability and Humic Acid Removal

**DOI:** 10.3390/membranes11090660

**Published:** 2021-08-27

**Authors:** Amirul Islah Nazri, Abdul Latif Ahmad, Mohd Hazwan Hussin

**Affiliations:** 1 School of Chemical Engineering, Engineering Campus, Universiti Sains Malaysia, Nibong Tebal 14300, Pulau Pinang, Malaysia; ain5721@gmail.com; 2 School of Chemical Sciences, Universiti Sains Malaysia, Gelugor 11800, Pulau Pinang, Malaysia; mhh@usm.my

**Keywords:** microcrystalline cellulose, polyethersulfone, composite membrane, lithium chloride/N,N-dimethylacetamide co-solvent, humic acid removal, water filtration

## Abstract

A novel polyethersulfone (PES)/microcrystalline cellulose (MCC) composite membrane for humic acid (HA) removal in water was fabricated using the phase inversion method by blending hydrophilic MCC with intrinsically hydrophobic PES in a lithium chloride/N,N-dimethylacetamide (LiCl/DMAc) co-solvent system. A rheological study indicated that the MCC-containing casting solutions exhibited a significant increase in viscosity, which directly influenced the composite membrane’s pore structure. Compared to the pristine PES membrane, the composite membranes have a larger surface pore size, elongated finger-like structure, and presence of sponge-like pores. The water contact angle and pure water flux of the composite membranes indicated an increase in hydrophilicity of the modified membranes. However, the permeability of the composite membranes started to decrease at 3 wt.% MCC and beyond. The natural organic matter removal experiments were performed using humic acid (HA) as the surface water pollutant. The hydrophobic HA rejection was significantly increased by the enhanced hydrophilic PES/MCC composite membrane via the hydrophobic–hydrophilic interaction and pore size exclusion. This study provides insight into the utilization of a low-cost and environmentally friendly additive to improve the hydrophilicity of PES membranes for efficient removal of HA in water.

## 1. Introduction

Membrane technologies are a promising alternative and have been continuously gaining popularity due to their intrinsic characteristics such as high selectivity, simple instrumental structure, environmental friendliness and low running investment [1]. Ultrafiltration membranes with pore size ranging from 0.001 to 0.1 µm are effectively used to remove suspended solids, bacteria, microorganisms, and viruses from surface water [2]. Polyethersulfone (PES) is one of the engineering polymers broadly used as ultrafiltration membrane materials due to its excellent chemical, mechanical, and thermal properties [3]. However, the main drawback of PES is associated with its non-optimal wettability and hydrophobicity characteristics [4]. The hydrophobic interaction between the humic acid (HA) solute and the membrane surface reduces water flux and, at the same time, increases the tendency of fouling.

HA is a dark brown, water-soluble, high-molecular-weight humic substance that originated from the natural decomposition of animals, microorganisms, and plant residues [5]. HA constitutes more than 50% of the natural organic matter (found in natural surface water, soils, and sediments) [6]. HA is a complex macromolecular polymer consisting of various functional groups, including carbonyl, quinonyl, carboxyl, and hydroxyl, joining together in a series of aromatic and alicyclic ring frameworks [7,8]. The presence of HA in municipal wastewater treatment plants may cause undesirable organoleptic properties in drinking water, such as brownish coloration and unpleasant taste in drinking water [9]. Additionally, HA can chelate various metal ions and other organic pollutants, thus facilitating the transportation and accumulation of toxic materials in aquatic systems [10]. In addition, HA acts as an essential precursor to the formation of disinfection by-products (DBPs) [11] when reacted with common chlorine-based disinfectants [12]. DBPs such as haloacetic acids [13] and trihalomethanes [14] are known to be highly carcinogenic, mutagenic, and teratogenic, which may lead to cancers, miscarriages, and nervous system complications [15]. Due to these concerns, HA has become global research focus mainly to reduce HA concentration in aqueous media, consequently controlling the formation of DBPs in potable waters [16].

The number of works on membrane modification is growing. The modification approach includes coating, plasma deposition, graft polymerization, and blending to either introduce additive materials on the membrane surfaces or incorporate them into the membrane matrix. Among these techniques, blending is preferred due to its convenient and mild operation conditions [17]. However, particle agglomeration is a major issue to be addressed, especially at a higher additive concentration. Particle agglomeration can negatively affect the morphology and the performance of the resulting membrane [18]. Ideally, the dispersion of hydrophilic particles must be uniform as this factor will significantly influence the performance and mechanical strength of a composite membrane prepared by the phase inversion method. To achieve such desired membrane properties, the selection of suitable types and compositions of hydrophilic particles must be looked at. Various types of inorganic materials with hydrophilic properties such as titanium dioxide [19], silica (SiO_2_), zirconia [20], graphene oxide (GO), carbon nanotubes [21], nanoclay [22], etc. have been used in the HA–water separation process. These materials were incorporated into the membranes to enhance the membrane’s hydrophilicity, pore structures, mechanical properties, and/or surface charges. Evidently, the addition of foreign particles in the polymer causes the structure and characteristics of the membrane to differ from its pristine form. For instance, the addition of green SiO_2_ from rice husk ash creates more finger-like structures, increases surface roughness, and increases the hydrophilicity of a polysulfone/SiO_2_ mixed matrix membrane (MMM) [23]. Meanwhile, Chai et al. synthesized a polysulfone MMM containing gum arabic (GA) and GO to enhance the membrane properties [24]. The MMM with 1.5% GA and 0.6% GO improved about 9.14% hydrophilicity, 32.22% porosity and pore size enlargement without compromising HA rejection.

Environmental protection has become extremely important in recent years to preserve and sustain a healthy and fresh environment. In parallel with that, green hydrophilic polymers, such as cellulose and its derivatives such as cellulose acetate (CA), CA pathlate, CA butyrate, cellulose nanocrystals (CNC), etc., have received much attention from researchers for water treatment applications [25,26,27]. Evangeline et al. synthesized an ultrafiltration membrane by blending PES with iron (III) oxide nanoparticles (Fe_2_O_3_) and CA [28]. The PES/CA/Fe_2_O_3_-modified composite membrane possessed enhanced hydrophilicity, porosity, and pore size and lower membrane resistance than the pristine PES and unmodified PES/CA blend membranes. The PES/CA/Fe_2_O_3_-modified membrane removed up to 70.3% fluoride with a maximum pure water flux (PWF) of 156 L m^−2^ h^−1^. Aside from the CA composite, nanocelluloses are receiving immense attention nowadays. Bai et al. had successfully removed 71.9% HA, 82.7% bovine serum albumin and 86% sodium alginate using a negatively charged, hydrophilic membrane by incorporating 2 wt.% of CNC into the PES matrix [29]. In another study, Bai et al. made a comparison between PES-modified membranes of CNC and cellulose nanofibrils (CNF) via the coating method [30]. They found that CNC exhibits better dispersibility and stronger negative charges than CNF. However, the CNF-modified membrane possesses a much rougher surface and higher PWF than the CNC-modified membrane. Ding et al. synthesized a lignin-CNF-modified PES membrane and reported that the lignin-CNF improves the morphology, thermostability, hydrophilicity and mechanical properties of the PES membrane [31].

Cellulose is regarded as an inexhaustible feedstock source since it is regularly regenerated by nature. Cellulose fabrication is estimated to be over 7.5 × 10^10^ tons annually [32]. Microcrystalline cellulose (MCC), one of the cellulose derivatives, is a renewable, environmentally friendly, and toxic-free material with high biocompatibility. MCC is used as binders in the pharmaceutical industry and anti-caking agent, thickener, texturizer, emulsifier, bulking agent, and fat substitute in the food industry due to its superior dry binding properties [33]. MCC is also used as potential reinforcement material for cement-based composites owing to its high surface area and strong mechanical properties, i.e., excellent tensile strength (greater than 7.5 GPa) and superior elastic modulus (approximately 150 GPa) [34]. The most significant part of MCC is its abundant hydroxyl (OH) groups, making it highly hydrophilic. It also has a high degree of crystallinity, between 55% and 80% [35,36,37]. These unique properties of MCC make it a promising alternative additive to improve the hydrophilicity and mechanical strength of the PES membrane. Despite all the attention given to cellulose and its derivatives, only a few reports have incorporated micro-sized cellulose into the PES membrane. For example, in 2020, Fatemeh et al. successfully embedded amine-functionalized MCC in the PES matrix [38]. The composite membrane possessed higher surface roughness, porosity, and hydrophilicity than the pristine PES. Although there are some studies on MCC implementation in the PES matrix, the effects of MCC on the separation performance of the PES/MCC composite membranes remain unclear. MCC has strong inter- and intra-hydrogen bonding, limiting its solubility in conventional membrane solvents [39]. It is, however, soluble in selected solvents, namely lithium chloride/N,N-dimethylacetamide (LiCl/DMAc) [40], sodium hydroxide/urea [41], dimethyl sulfoxide/tetrabutylammonium fluoride [42], ionic liquids [43], and N-methyl morpholine oxide [44]. The LiCl/DMAc co-solvent was chosen for this study because DMAc is a commonly used solvent in membrane fabrication while LiCl is a pore former [45]. The dissolution mechanism of cellulose in the LiCl/DMAc co-solvent has been reported by several researchers [46,47]. However, to the best of our knowledge, there is no report on the reaction mechanism pathway between the dissolute MCC in LiCl/DMAc co-solvent and PES.

This study is intended to report the hydrophilic composite membrane fabricated by integrating PES and MCC in the LiCl/DMAc co-solvent via the blending technique using a phase inversion method. MCC was loaded at varying concentrations to comprehensively elucidate its influence on the viscosity of casting solutions, the microstructure, water contact angle, surface chemistry, and the mechanical properties of the composite membrane. In addition, the performance of the PES/MCC composite membrane in terms of PWF and HA rejection in relation to its properties was investigated.

## 2. Materials and Methods

### 2.1. Materials

Polyethersulfone (PES; Ultrason E6020P) with a molecular weight of 58,000 g mol^−1^ was purchased from BASF. Microcrystalline cellulose (MCC; particle size: 20 μm), humic acid (HA; technical grade) and sodium hydroxide (NaOH) were supplied by Sigma-Aldrich, while N,N-dimethylacetamide (DMAc) was obtained from Acros Organic. Lithium chloride (LiCl) was purchased from Merck.

### 2.2. Preparation of MCC Solution

MCC solution was prepared using a modified method of Wan et al. [48]. A co-solvent solution of 8 wt.% LiCl in DMAc was prepared according to predetermined ratios (Table 1) by dissolving an appropriate amount of LiCl powder in the weighed amount of DMAc. The mixture was continuously stirred for 2 h at 110 °C until a homogeneous solution was formed. Next, the desired amount of MCC powder was slowly added into the co-solvent under vigorous magnetic stirring at the same temperature for another 50 min. Finally, the suspension was stirred overnight at 30 °C to obtain a homogeneous MCC solution.

### 2.3. Membrane Fabrication

Membranes were prepared by the non-solvent-induced phase separation method. First, PES flakes were dried at 70 °C in a vacuum oven for at least 24 h. In addition, the prepared MCC solution was sonicated at 60 °C for 1 h before mixing with PES flakes. A casting solution of 17.25 wt.% of PES was prepared according to predetermined ratios (Table 1) by slowly dissolving the desired amount of PES flake in the prepared MCC solution. The mixtures were continuously stirred for 24 h at 60 °C until a homogeneous solution was formed. The solution was degassed in an ultrasonic bath for 1 h at 60 °C to remove air bubbles. Subsequently, the solution was cast on a clean glass plate by a film applicator. The casting speed was set to 20 mm s^−1^ at room temperature, and the casting knife thickness was 200 μm. The thin casted film was left in the air for 45 s and subsequently immersed in the distilled water coagulation bath for 24 h. Finally, the membranes were rinsed in tap water and stored in distilled water before usage.

### 2.4. Preparation of HA Solution

HA solution was prepared by dissolving 0.05 g HA in 1 L of distilled water. A few drops of 1 M NaOH were introduced into the solution and then sonicated for 1 h. Consequently, the solution was filled with distilled water until it reached the 1 L mark and continuously stirred for at least 24 h. To further aid the HA dissolution, the pH was kept around 7.7 by adding drops of 1 M NaOH into the HA solution.

### 2.5. Characterization

#### 2.5.1. Viscosity of Casting Solutions

The rheological properties of PES/DMAc and PES/MCC/LiCl/DMAc casting solutions were measured by a programmable rheometer (Brookfield DV-III Ultra). The temperature was maintained at 30 °C by a water bath. The measurements of the shear viscosity (cP) and shear rate (s^−1^) were obtained at the torque reading between 10% and 100% by varying the rotating speed of the spindle from 0 rpm to 250 rpm. The zero-shear viscosity was determined using the Origin Pro 2019 software.

#### 2.5.2. Membrane Morphology

The morphology of the membrane was studied using a scanning electron microscope (SEM; Hitachi S-3000N, Hitachi Ltd., Tokyo, Japan). To obtain a membrane’s cross-sectional images, the membrane was first fractured in liquid nitrogen and then attached to the sample holder by carbon tapes before coating with a thin layer of gold using a sputter coater. The acquired images were processed and analyzed using the ImageJ version 1.43u software (Java™ Platform SE Binary) to estimate the pore size of the fabricated membranes.

#### 2.5.3. Membrane Surface Chemistry

The functional groups and chemical bonding present in the commercial MCC powder and the fabricated membranes were determined using Fourier transform infrared spectroscopy in the attenuated total reflection mode (FTIR-ATR, Thermo Scientific Nicolet Nexus 670, Waltham, MA, USA). All samples were scanned over the wavenumber ranging from 650 cm^−1^ to 4000 cm^−1^.

#### 2.5.4. Membrane Mechanical Properties

The tensile tests of the membranes were carried out at room temperature using a Tensile Tester Machine (Instron, Model 3366, Norwood, MA, USA). Membrane samples were cut according to ASTM D638 Type V dumbbell shape. The membrane thickness was determined by a hand-held thickness gauge (Mitutoyo, No. 7331, Kawasaki, Japan) and reported as the mean of five measurements taken over the length of the test specimen. The gauge length was adjusted to 20 mm at the start of each test. The ends of the test specimen were mounted and clamped by using steel grip jaws. The measurements were conducted with a crosshead speed of 2 mm min^−1^.

The results were recorded for force (F) as a function of the sample elongation (∆L = L − L_0_), where L_0_ was the original length of the test specimen. The stress against strain curve was constructed from the results obtained in the test, and two properties were determined from the curve: (1) tensile strength and (2) elongation-at-break.

#### 2.5.5. Membrane Water Contact Angle

The water contact angle (WCA) is an indicator of membrane surface wettability. The WCA of the fabricated membranes was measured using a goniometer (Rame-Hart 250 F-1, Succasunna, NJ, USA) using the sessile drop method. A 6 μL water droplet of the micro-syringe was dropped onto the fabricated membrane surface. To minimize the error, WCA at five different points of each sample was measured at room temperature, and the average WCA was reported.

### 2.6. Membrane Flux and HA Rejection Evaluation

**Figure 1:**
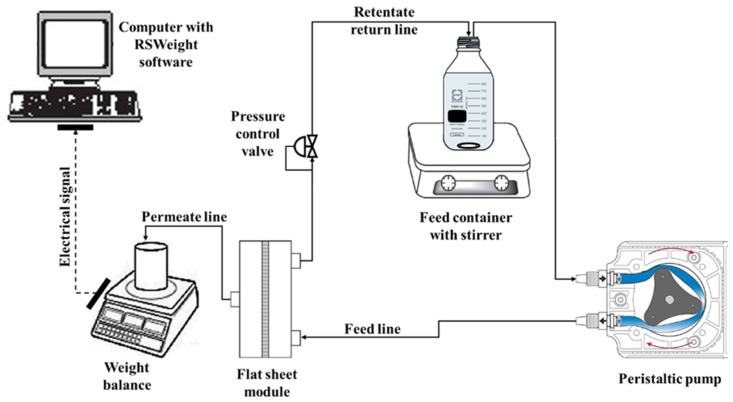
Experimental setup for membrane permeation using the crossflow filtration configuration.

The PWF and HA rejection was tested by a standard filtration cell for flat sheet membranes using a crossflow configuration setup, as shown in Figure 1. An effective membrane area of 44.4 cm^2^ was used. All the experiments were carried out with the feedstock solutions stirred at 500 rpm at room temperature. The volumetric flow rate of the feed solution was maintained at 400 mL min^−1^ during the whole experiment unless stated otherwise. Each membrane was pre-compressed at 1.5 bar for 1 h to stabilize the filtration before all the measurements [49]. Subsequently, the pressure was changed to 1 bar, and the process was left for 10 min to achieve a steady filtration rate at the desired pressure. Water permeation experiment was run at a pressure of 1 bar for 1 h and the PWF was calculated according to Equation (1):

(1) $$J_{\mathrm{PWF}}=\frac{Q}{A\times T},$$

where J_PWF_ is the distilled water permeation flux (L m^−2^ h^−1^), *Q* is the volume of permeation (L), *A* is the effective membrane area (m^2^), and *T* is the filtration time (h).

HA was used as a model for the rejection test. After the PWF measurement, the feed solution was replaced by 50 mg L^−1^ HA solution prepared earlier. The HA rejection test was conducted at 1 bar for 1 h. The HA concentration of the permeate and the feed solution were measured using a UV–Vis spectrophotometer (UV-Vis Cary 60) at a wavelength of 254 nm [50]. The membrane rejection was calculated as follows:

(2) $$\mathrm{Rejection}\;[\%]=\left[1-\left(\frac{C_{P}}{C_{F}}\right)\right]\times 100,$$

where *C_P_* and *C_F_* are the HA concentration in the permeate and the feed solution (mg L^−1^), respectively. After HA filtration, the membrane was washed using distilled water at a volumetric flow rate of 600 mL min^−1^ for 15 min without any applied pressure. The PWF was measured again under the same conditions as the first PWF experiment.

## 3. Results and Discussion

### 3.1. Surface Functional Groups

**Figure 2:**
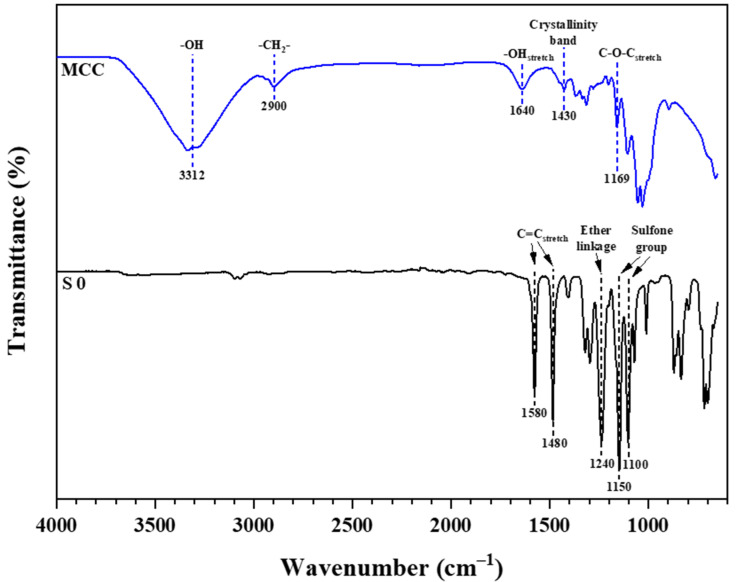
FTIR spectrum of commercial MCC and the pristine PES membrane (S0).

FTIR spectroscopy studies were carried out to characterize the membrane’s surface functional groups. FTIR spectra of commercial MCC powder and fabricated pristine PES membrane are shown in Figure 2. The results for commercial MCC powder showed a similar infrared pattern to the pure cellulose and CNC as reported in the literature [30,37,51]. This similarity indicates that the MCC has the same chemical composition as pure cellulose and its other derivatives. The broad absorption band from 3300 cm^−1^ to 3400 cm^−1^ corresponds to the stretching vibration of OH groups, while the following absorption peak at 2900 cm^−1^ is correlated with CH_2_ groups [52]. The absorption peak at 1640 cm^−1^ is due to the absorbed water molecules resulting from a strong interaction between cellulose and water. Besides, the absorption band at 1430 cm^−1^ is assigned to symmetric CH_2_ bending vibration. This band is also known as the “crystallinity band,” which illustrates cellulose’s degree of crystallinity. The bands at 1169 cm^−1^ and 895 cm^−1^ are assigned to C–O–C stretching (pyranose ring ether) and C–H rock vibration of cellulose (anomeric vibration of β-glucosides), respectively.

**Figure 3:**
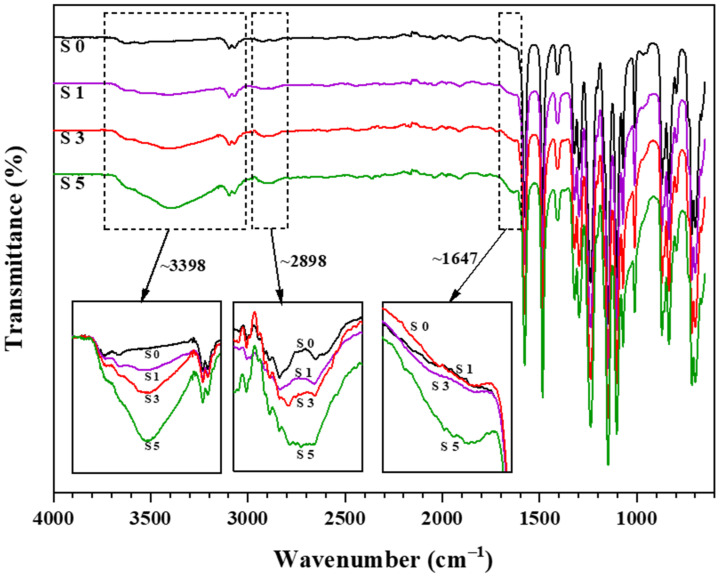
FTIR spectra of pristine PES (S0) and MCC-incorporated PES membranes at 1 wt.% MCC (S1), 3 wt.% MCC (S3), and 5 wt.% MCC (S5) loading.

The FTIR spectrum of the fabricated pristine PES membrane (S0) shows absorption peaks at 1580 cm^−1^ and 1480 cm^−1^, representing C=C stretching vibration of benzene rings. The peak at 1240 cm^−1^ correlates to ether linkage between phenyl groups. In addition, the absorption bands at both 1150 cm^−1^ and 1100 cm^−1^ correspond to the sulfone group in the PES base structure. All the peaks obtained are similar to those reported by Athira et al. [3]. To study the effect of different MCC loadings on the fabricated membranes, FTIR spectra of PES/MCC composite membranes were analyzed. The spectra of 1 wt.% MCC (S1), 3 wt.% MCC (S3), and 5 wt.% MCC (S5) incorporated PES membranes, together with the membrane S0, are represented in Figure 3.

The spectra of the PES/MCC composite membranes also showed a similar spectrum as the pristine PES membrane, especially from 1500 cm^−1^ to 500 cm^−1^. This behavior indicates that the composite membranes could retain the PES characteristics even with the inclusion of MCC. However, significant changes were observed at the peak region ~3398 cm^−1^, ~2898 cm^−1^, and ~1647 cm^−1^, representing the OH groups, CH_2_ groups, and water absorbed, respectively. These peaks are similar to what is being recorded for MCC powder, as shown in Figure 2, indicating the success of MCC incorporation into the PES matrix. MCC dissolution in the LiCl/DMAc co-solvent broke the hydrogen bonds within the MCC structure [53]. The OH groups of the dissolved MCC were then able to form new hydrogen bonds with PES. The hydrogen bonds are physically connected to the MCC with PES, making the PES/MCC composite membranes possess MCC properties while sustaining the PES characteristics. Furthermore, increasing the MCC loadings intensifies all the three peaks mentioned. This indicates that more OH is attached to the composite membranes. As a result, the surface hydrophilicity of the membranes was enhanced.

### 3.2. Casting Solution Viscosity

**Figure 4:**
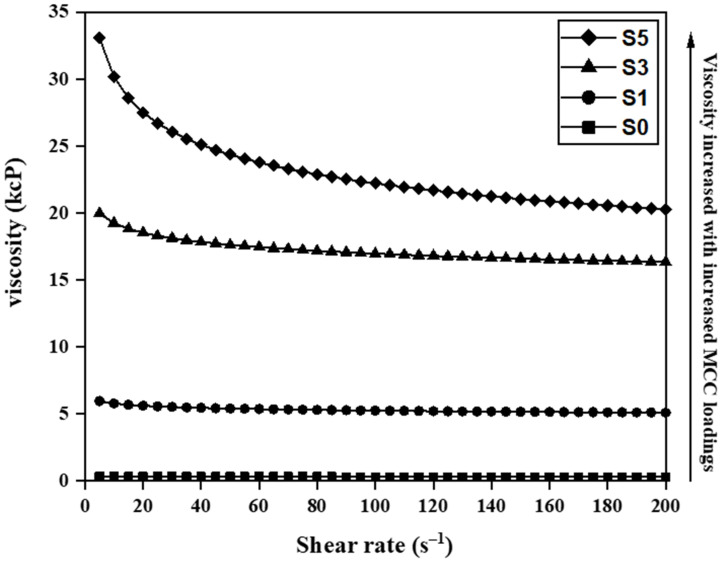
Viscosity of casting solutions for pristine PES (S0) and MCC-incorporated PES membranes with 1 wt.% (S1), 3 wt.% (S3), and 5 wt.% (S5) MCC loadings.

The viscosity of the casting solutions of pristine PES and PES/MCC composite membranes can be found in Figure 4. Pristine PES (S0) recorded a viscosity of 300 cP while the composite membrane recorded increasing viscosity up to 30,000 cP with increasing MCC loadings. The high viscosity of the casting solution for the composite membranes has resulted from the interaction between LiCl, DMAc, and PES. Zheng et al. reported viscosity increment by LiCl addition due to strong interaction between LiCl, DMAc and the polymer [45]. Besides that, the trend of increasing viscosity with increasing MCC loadings can be associated with the interactions between LiCl, MCC, and PES. Zhang et al. claimed that chlorine ions in the LiCl/DMAc co-solvent would form hydrogen bonds with OH groups within MCC [54]. The chlorine–MCC complex will then interact with PES via hydrogen bonds between the MCC’s OH and oxygen groups (and/or carbonyl groups) of the PES polymer [55]. Higher MCC loading caused more OH groups to readily bond with the PES and led to the trend of increasing viscosity.

### 3.3. Membranes Morphology and Dope Viscosity

**Figure 5:**
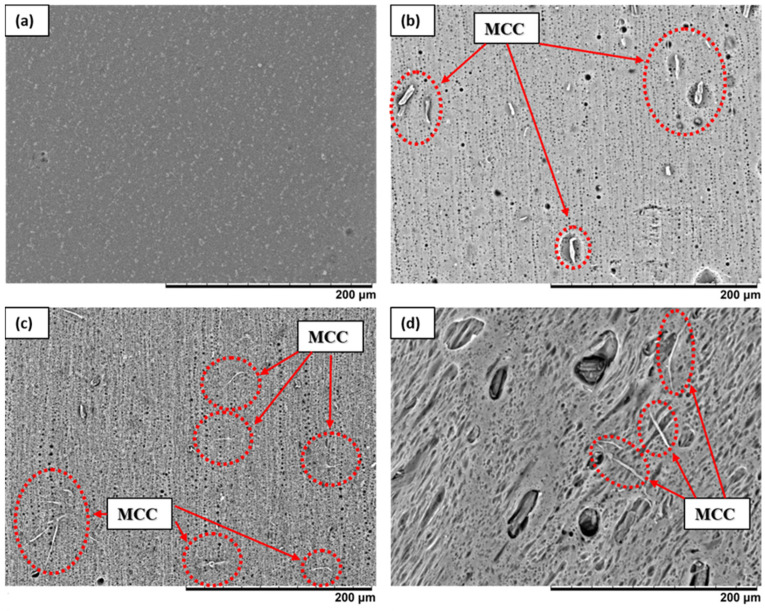
SEM for the top surface image of (**a**) pristine PES and MCC-incorporated PES membranes with (**b**) 1 wt.% MCC, (**c**) 3 wt.% MCC, and (**d**) 5 wt.% MCC at 500× magnification.

**Figure 6:**
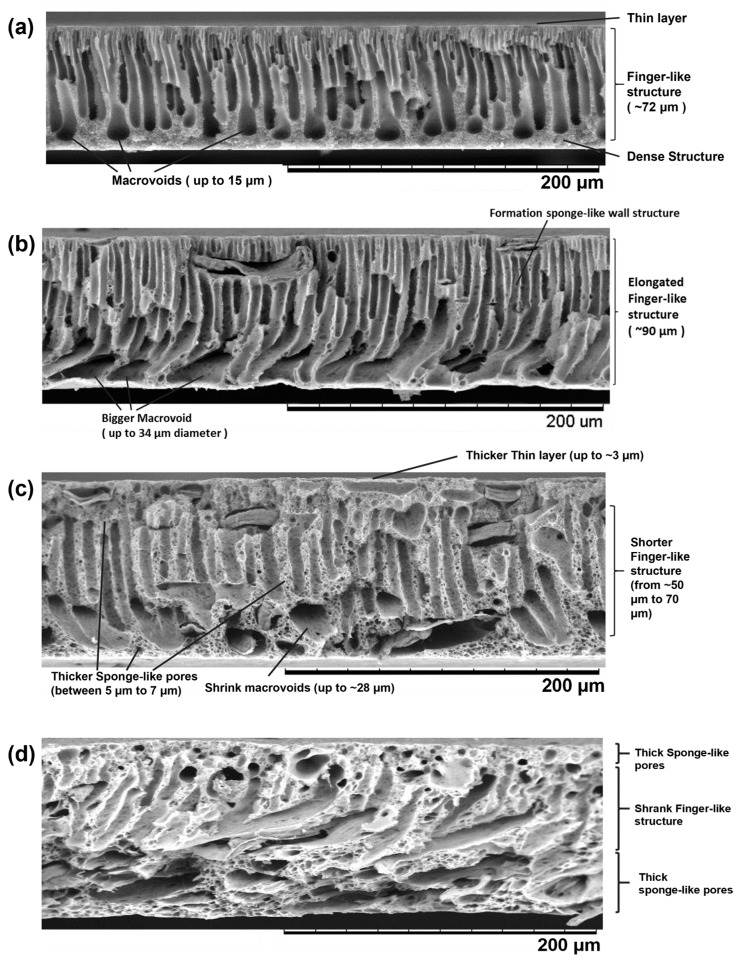
SEM for cross-sectional images of (**a**) pristine PES and MCC-incorporated PES membranes with (**b**) 1 wt.% MCC, (**c**) 3 wt.% MCC, and (**d**) 5 wt.% MCC at 500× magnification.

The surface and cross-section morphology of the membranes fabricated in this study are presented in Figure 5 and Figure 6, respectively. In general, all fabricated membranes possess an asymmetric membrane structure, i.e., a thin dense top layer supported by finger-like pores in the middle and macrovoid pores at the bottom. The presence of MCC on the surface of the composite membranes S1, S3, and S5 is clear, as shown by the red circles in Figure 5. This indicates that the MCC was successfully incorporated into the PES matrix. The effect of various MCC loadings on the pore structures and sizes is apparent. An enlargement of pore size as a function of MCC loading is visible on the surface of the PES/MCC composite membranes, as shown in Figure 5.

Similarly, the cross-section morphology of the membrane reveals that the size of the macrovoid pores increased, and the finger-like pores elongated toward the bottom part of the composite membrane S1. This is because the presence of MCC in the casting solution increased the phase inversion rate due to its hydrophilic property, which accelerates the velocity of water diffusion into the casting solution [38]. Besides, there is a noticeable formation of sponge-like pores on the membrane walls due to the presence of LiCl in the casting solution. An identical result was reported by Mansourizadeh et al. when adding a high concentration of LiCl, i.e., 4 wt.%, induced the formation of a sponge-like structure [56].

Different pore structures were recorded when using 3 wt.% and 5 wt.% MCC loading. Thicker sponge-like pores were observed on both membranes, with composite membrane S5 being the thickest. The thickness of both membranes’ active thin top layer increased with the increased amount of MCC used. In addition, the macrovoid and finger-like pores shrank at higher loading of MCC. Similar changes in pore structure were reported by Zhang et al. [57]. In their study, they altered the viscosity of the casting solution by manipulating the amount of polysulfone added to the casting solution. They obtained thick-skinned top-layer membranes with a less porous surface and reduced numbers of suppressed finger-like pores as a result of the low phase inversion process due to the high viscosity of the casting solutions. Similarly, both S3 and S5 membranes had a high viscosity of 18,000 cP and 22,000 cP, respectively. In this case, viscosity became the dominant factor determining the rate of phase inversion, outweighing the hydrophilicity that MCC offers. Instead of having a fast rate of phase inversion, the casting solution experiences slower phase inversion due to its highly viscous character. During the phase inversion process, the inflow of the non-solvent was hindered, causing less water to be exchanged into the casting solution [58]. As a result, the macrovoids became narrower, the sponge-like pore growth expanded, and the finger-like pores shrank.

### 3.4. Mechanical Properties of Fabricated Membranes

**Figure 7:**
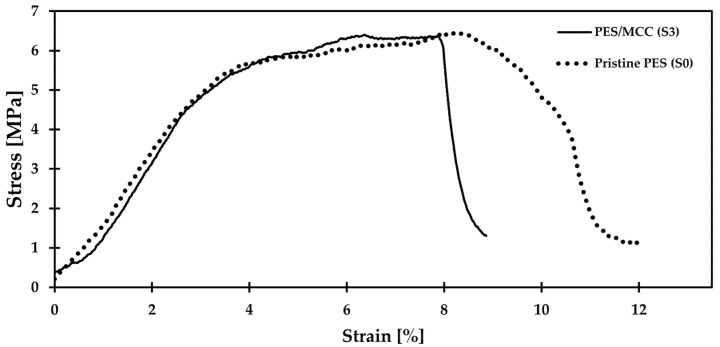
Stress–strain behavior of pristine PES and PES/MCC composite membranes at room temperature.

Figure 7 represents the stress–strain graph comparing the tensile behavior of both pristine PES and PES/MCC composite membranes. The tensile strength and elongation-at-break for both membranes were extracted from the figure and tabulated in Table 2. This test used membranes S0 and S3 to represent the pristine and composite membranes, respectively. The result shows a significant decrease in elongation-at-break and only a tiny decrease in tensile strength of the PES/MCC composite membrane compared to the pristine PES membrane. The reduced elongation shows that the composite membrane became brittle due to the interaction of MCC with the PES matrix. PES is pseudoplastic and possesses the ductility properties of a polymer [59], while MCC has a highly crystalline region. The interaction between MCC and PES led to the reduced elasticity and increased brittleness of the composite membrane due to the presence of micro-crystals within the PES matrix. Meanwhile, the slight decrease in tensile strength could be due to the aggregation of MCC when a high concentration of MCC was used in the casting solution. Similar behavior was reported by J. Lv et al. when the tensile strength of cellulose diacetate/CNC composite membrane decreased when more than 3 wt.% CNC were used [60]. The MCC aggregation may cause interaction among MCCs or PES to take over the PES/MCC interaction [61]. Furthermore, the MCC agglomeration increases the micro-structural inhomogeneity and acts as the weak link, resulting in reduced tensile strength of the composite membrane [60].

### 3.5. Hydrophilicity Analysis of Fabricated Membranes

**Figure 8:**
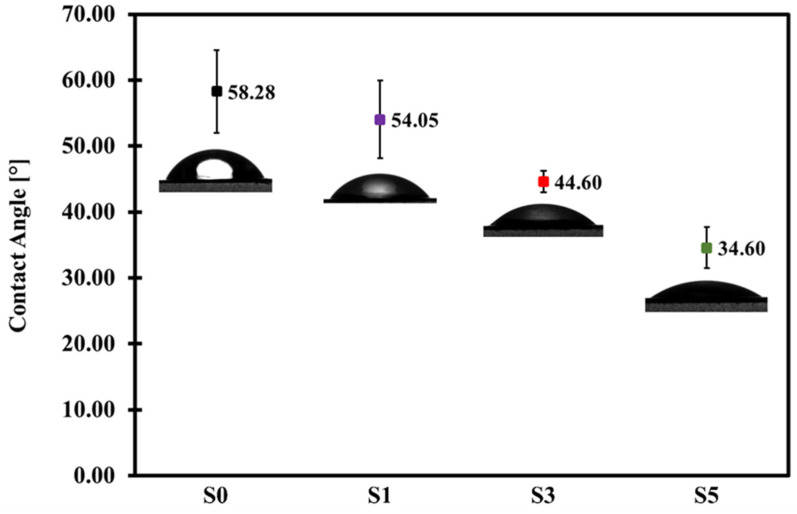
Static water contact angle (WCA) of pristine PES (S0), and MCC-incorporated PES membranes with 1 wt.% MCC (S1), 3 wt.% MCC (S3), and 5 wt.% MCC (S5).

Figure 8 summarizes the static WCA of the fabricated membranes. The pristine PES membrane exhibited a WCA value of 58.28°. The composite membranes with 1 wt.%, 3 wt.%, and 5 wt.% MCC loadings have a WCA of 54.05°, 44.60°, and 34.60°, respectively. The trend shows improved hydrophilicity of the PES/MCC composite membranes by increasing the MCC loading. In general, a high amount of LiCl in the casting solutions will induce polymer crystallization and increased membrane surface roughness, leading to increased hydrophobicity [58]. However, the presence of MCC in the casting solution improved the hydrophilicity of the composite membranes by enhancing their pore structures (Figure 5 and Figure 6) and chemically introduced the OH groups onto the membrane surface (Figure 3). The presence of OH groups will cause any air gaps on the membrane surface to have a high tendency to be filled with water due to their ability to attract water, thus promoting membrane wetting. As the amount of MCC increases, the amount of OH groups on the membrane surface also increases. As a result, membrane wetting increases. Therefore, it can be concluded that incorporating hydrophilic MCC containing a high amount of OH groups improved the hydrophilic nature of the PES membrane significantly.

### 3.6. Permeability and Separation Performance of Fabricated Membranes

**Figure 9:**
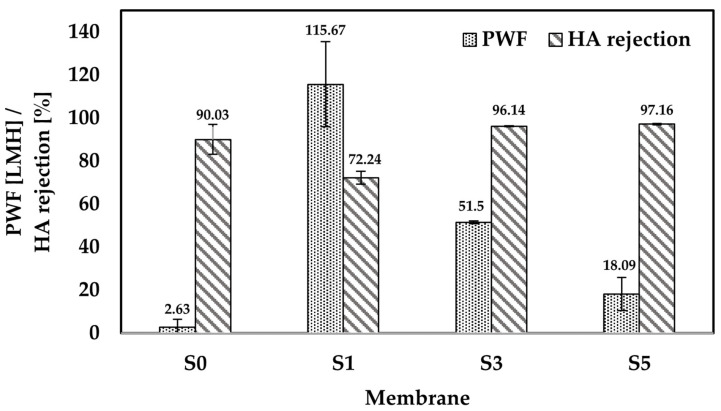
Performance tests in terms of PWF and HA rejection for the pristine PES membrane without any pore former (S0) and the composite membranes S1, S3, and S5 after 1 h filtration time.

Crossflow filtration processes were performed to investigate the water permeability and HA rejection of the fabricated membranes. The results for the PWF within a 1 h period are shown in Figure 9. Pristine PES membrane (S0) containing 17.25 wt.% PES recorded a low average PWF of 2.63 L m^−2^ h^−1^ (LMH) at 1 bar operating pressure. This is due to the small surface pores and low surface hydrophilicity of the membrane, which mostly negates the penetration of the water molecules through the membrane pores. Similar behavior was recorded by Evangeline et al. for their 18 wt.% PES flat sheet membrane where the permeability recorded for the membrane was 3 L m^−2^ h^−1^ bar^−1^ [LMHB] [28]. The low value of water permeation is due to the absence of a pore former, which led to the formation of small membrane pores. In contrast, Zhang et al. reported a higher water permeation of 32 LMHB for 15 wt.% PES membrane in the presence of 2 wt.% polyvinylpyrrolidone as a hydrophilic pore former [49]. The smaller amount of PES used compared to our study, together with the presence of a pore former, led to bigger membrane pores, which allowed more water molecules to pass through the membrane.

The MCC loadings of 1 wt.%, 3 wt.%, and 5 wt.% increased the PWF to 115 LMH, 51.5 LMH, and 18.09 LMH, respectively. The synergy effect between the presence of OH group and pore size led to the significant increase in PWF of the composite membranes compared to the pristine PES membrane. The substantial changes in the flux are mainly attributed to the hydrophilicity and the pore size of the composite membranes. In terms of surface hydrophilicity, the hydrophilic MCCs could enhance the transport rate of water molecules due to the large OH groups. The increased hydrophilicity of the composite membranes led to the attraction of the water molecules by the OH groups on the membrane surface. Meanwhile, the increased surface pore size of these membranes led to a higher volume of water molecules to pass through the membranes.

However, a decrease of PWF was recorded at MCC loading higher than 1 wt.% (composite membranes S3 and S5). The significant increase in viscosity of the casting solution has directly affected the membrane pore structure. The composite membrane S3 has a thicker top active layer compared to composite membrane S1, as shown in Figure 6. In addition, a shorter finger-like structure with shrank macrovoids was also recorded for the composite membrane S3. Such pore structures cause resistance for water molecules to penetrate through the membrane.

Further reductions in PWF were recorded at 5 wt.% MCC loading. The high viscosity casting solution, i.e., 25,000 cP, of the composite membrane S5 (Figure 4**)** led to the formation of thicker sponge-like pores than the composite membrane S3 at the top and bottom parts of the membrane (Figure 6). The growth of sponge-like pores causes the finger-like pores and macropores to reduce in number and size. These unfavored pore structures provide higher resistance than membrane S3 for water molecules to penetrate through the membrane, leading to the undesired low PWF [57].

The HA rejection for all membranes tested is shown in Figure 9. Feed solutions with a concentration of 50 mg L^−1^ HA were used in the filtration process. The high rejection of 90.03% HA was recorded for the pristine PES membrane due to the small surface pores of the membrane. However, the significantly low PWF, i.e., 2.63 LMH, of the membrane is not favorable. With 1 wt.% MCC loading, the PWF flux increased significantly to 115.67 LMH due to the elongated finger-like pores down to the bottom part of the membrane. The increased flux led to the reduced HA rejection of 72.24% by the composite membrane S1. The rejection is higher than previously reported, which recorded only 45% HA removal using MMM embedded with multi-walled carbon nanotubes [21]. HA contained a broad molecular size distribution, including small molecules below 1 kDa [62]. The small molecules would easily pass through the membrane pore structure, leading to reduced HA rejection.

On the other hand, composite membranes S3 and S5 showed a significant increase in HA rejection compared to the pristine PES membrane. Both composite membranes S3 and S5 recorded a much higher HA rejection than the pristine PES membrane with 96.14% and 97.16% HA rejection, respectively. However, both membranes suffered a lower PWF than composite membrane S1, which is 51.5 LMH for S3 and 18.09 LMH for S5. The result for composite membrane S3 is acceptable due to a significant increase in both PWF and HA rejection relative to the pristine PES membrane. Meanwhile, composite membrane S5 showed a substantial loss in PWF from composite membrane S3 without any significant increase in HA rejection. The physicochemical properties of the composite membranes are responsible for the improved HA rejection. Both composite membranes S3 and S5 showed the formation of a thicker top active layer than the composite membrane S1, as a result of low phase inversion rate due to the high viscosity of the casting solutions. Furthermore, both membranes possessed a high degree of hydrophilic properties (Figure 8), which induced a dense and stable water hydration layer on top of the membranes during the filtration process [29]. Moreover, the high hydrophilicity of these membranes reduced the hydrophobic attraction between the membrane surface and HA, which resulted in improved HA rejection. These combined properties aided the separation of HA while letting the water molecules to pass through. Hence, it may be deduced that hydrophilicity and pore sizes played a vital role in HA removal.

### 3.7. Comparison with Other Studies

The uniqueness of this work focuses on improving the hydrophobic PES membrane performance by adopting an eco-friendly additive MCC in the PES matrix with the assistance of the LiCl/DMAc co-solvent system. Generally, the incorporation of MCC into the PES polymer matrix was successful, and the composite membrane displayed better hydrophilicity, water flux and HA rejection than the pristine PES membrane. Therefore, the adoption of the eco-friendly MCC marked a noteworthy development toward PES membrane enhancement. Table 3 shows a comparison in membrane performance between this study and the literature. As shown in the table, the presence of hydrophilic additive contributes to the membrane performance in terms of water flux and solute rejection. The membranes stated in the table (including this study) recorded increased water permeability, albeit at a different extent due to the different formulations and additives used.

Our study recorded an enhanced water permeability of 51.50 LMHB, which is significantly higher than that reported by Evangeline et al. [28] and Zha et al. [27]. Meanwhile, Chai et al. reported 56.68 LMH water flux at 4 bar operating pressure and 96.34% HA rejection when using an MMM embedded with GO and AG [24]. The results are comparable with the results obtained in this work for the composite membrane with 3 wt.% MCC loading, i.e., 51.5 LMH with 96.14% HA rejection. A few studies have reported much higher water permeability than our studies, such as 485 LMHB [29] and 692 LMHB [31], when adopting CNC and LCNF in the PES matrix in the presence of a PVP hydrophilic pore former. This is due to the synergetic effect between the hydrophilic nanoparticle and the hydrophilic pore former used in the casting solution, which further increased the phase inversion rate and the membrane pore size. Nonetheless, our study demonstrated minimal trade-off effect between water flux and HA rejection as the water flux recorded a severalfold increment and improved HA rejection compared to pristine PES membrane.

## 4. Conclusions

In this study, MCC-incorporated PES composite membranes were fabricated by the phase inversion approach using the LiCl/DMAc co-solvent. The succession of MCC incorporation into the PES matrix shown in FTIR analysis indicates good compatibility between PES and MCC to form bonds to each other in the presence of the LiCl/DMAc co-solvent. In addition, the changes in the mechanical properties of both pristine PES and PES/MCC composite membranes from ductile to brittle further proved the point. SEM analysis showed that enhanced pore structure was obtained in the presence of up to 3 wt.% MCC. The hydrophilicity of MCC increases the phase inversion rate and leads to a bigger surface pore, a more elongated finger-like pore, and a bigger macro-size pore. Besides that, the incorporation of MCC improved the PES membrane’s hydrophilicity based on the reduced WCA, down to 34.6°, obtained for the PES/MCC composite membranes resulting from OH group availability on the membrane surface and the increase in their surface pore size. The modified membrane showed better separation performance toward both PWF and HA rejection. Based on the filtration evaluation, the presence of 3 wt.% of MCC loading composite membrane resulted in the formation of an optimum PES composite membrane with an improved PWF of 51.34 LMH (20 times higher than pristine PES membrane) and a high HA rejection of 96.14% (6.8% increase from pristine PES membrane) at 1 bar operating pressure. The comparison made with other studies showed that the results obtained in this study are competitive with those of the membranes loaded with GO and CNC additives.

## Figures and Tables

**Table 1:** Composition of the prepared casting solutions (total mass of casting solution = 30 g).

Sample	PES ^1^ [g]	MCC ^2^ [g]	Co-Solvent	
			LiCl ^3^ [g]	DMAc ^4^ [g]
S0	5.175	0	0	24.825
S1	5.175	0.3	1.962	22.563
S3	5.175	0.9	1.914	22.011
S5	5.175	1.5	1.866	21.459

^1^ PES maintained at 17.25 wt.%. ^2^ MCC varied for 0, 1, 3, and 5 wt.%. ^3^ LiCl calculated from 8 wt.% of the total amount of co-solvent used. ^4^ DMAc calculated from 92 wt.% of the total amount of co-solvent used.

**Table 2:** Average tensile strength and elongation-at-break for pristine PES and PES/MCC composite membranes.

Membrane	Tensile Strength [MPa]	Elongation-at-Break [%]
Pristine PES (S0)	6.57 ± 1.52	11.88 ± 5.72
PES/MCC (S3)	5.71 ± 0.89	7.16 ± 1.71

**Table 3:** Performance comparison between different optimized composite membranes reported in the literature and this study.

Membrane	Configuration		Operating Pressure	PWF ^1^	Water Permeability ^2^	Solute Rejection	Ref.
	Membrane	Filtration					
PES/CA (18/2 wt.%)	FS	Dead-end	2.94 bar	15.00 LMH	Improved to 5.10 LMHB	Fluoride—8%	[28]
PES/CAP/PVP (12.8/3.2/2 wt.%)	FS	Crossflow	3.45 bar	400.00 LMH	Improved to 115.94 LMHB	Non-skim milk—99%	[26]
PES/CAB/PVP (27.26/1.74/1 wt.%)	HF	Inside-out	6.89 bar	2.60 LMH	Improved to 0.38 LMHB	Benzene—99.6% Toluene—98.3% Octanoic acid—99.6% Hexanoic acid—99.6%	[27]
PES/LCNF/PVP (18/0.3/1.2 wt.%)	FS	Dead-end	1.00 bar	692.30 LMH	Improved to 692.30 LMHB	BSA—95%	[31]
PES/CNC/PVP (15/0.15/2 wt.%)	FS	Dead-end	2.78 bar	190.00 LMH	Improved to 68.35 LMHB	BSA—96.2%	[49]
PES/CNC/PVP (15/2/3 wt.%)	FS	Dead-end	0.60 bar	291.00 LMH	Improved to 485.00 LMHB	HA—71.9% BSA—82.7% NaAlg—85%	[29]
PSf/GO/GA (18/2/3 wt.%)	FS	Dead-end	4.00 bar	58.68 LMH	Improved to 14.67 LMHB	HA—96.34%	[24]
PES/MCC (17.25/3 wt.%)	FS	Crossflow	1.00 bar	51.50 LMH	Improved to 51.50 LMHB	HA—96.14%	This study

^1^ PWF refers to the water permeation rate for operation pressure specified in the literature, i.e., L m^−2^ h^−1^ (LMH). ^2^ Water permeability refers to the water permeation rate for 1 bar operating pressure, i.e., L m^−2^ h^−1^ bar^−1^ (LMHB). PES = polyethersulfone, PSf = polysulfone, PVP = polyvinylpyrrolidone, CA = cellulose acetate, CAP = cellulose acetate phthalate, CAB = cellulose acetate butyrate, LCNF = lignin cellulose nanofibril, CNC = cellulose nanocrystal, GO = graphene oxide, GA = gum Arabic, FS = flat sheet, HF = hollow fiber, BSA = bovine serum albumin, HA = humic acid.

## Data Availability

No new data were created or analyzed in this study. Data sharing is not applicable to this article.
